# Genetic Association Analysis of ATP Binding Cassette Protein Family Reveals a Novel Association of *ABCB1* Genetic Variants with Epilepsy Risk, but Not with Drug-Resistance

**DOI:** 10.1371/journal.pone.0089253

**Published:** 2014-02-21

**Authors:** Shabeesh Balan, Sumitha Prameela Bharathan, Neetha Nanoth Vellichiramal, Sanish Sathyan, Vijai Joseph, Kurupath Radhakrishnan, Moinak Banerjee

**Affiliations:** 1 Human Molecular Genetics Laboratory, Rajiv Gandhi Center for Biotechnology, Trivandrum, Kerala, India; 2 R. Madhavan Nayar Center for Comprehensive Epilepsy Care, Sree Chitra Tirunal Institute for Medical Sciences and Technology, Trivandrum, Kerala, India; 3 Department of Medicine, Memorial-Sloan Kettering Cancer Center, New York, New York, United States of America; University of Technology Sydney, Australia

## Abstract

Epilepsy constitutes a heterogeneous group of disorders that is characterized by recurrent unprovoked seizures due to widely different etiologies. Multidrug resistance remains a major issue in clinical epileptology, where one third of patients with epilepsy continue to have seizures. Role of efflux transporters in multidrug resistant epilepsy has been attributed to drug-resistant epilepsy although, with discrepant observation in genetic studies. These discrepancies could be attributed to variety of factors such as variable definition of the anti-epileptic drug (AED)-resistance, variable epilepsy phenotypes and ethnicities among the studies. In the present study we inquired the role of multidrug transporters *ABCB1* and *ABCG2* variants in determining AED-resistance and susceptibility to epilepsy in three well-characterized cohorts comprising of mesial temporal lobe epilepsy with hippocampal sclerosis (MTLE-HS) (prototype for AED-resistant epilepsy); juvenile myoclonic epilepsy (JME) (prototype for AED-responsive epilepsy); and healthy non-epileptic controls, in 738 subjects of Malayalam speaking south Indian ancestry. *ABCB1* and *ABCG2* variants were not found to be associated with drug resistance when AED-resistant and AED-responsive cohorts were compared. However, a significant association was observed between *ABCB1* (C3435T) rs1045642 and risk of having epilepsy (MTLE-HS and JME pooled cohort; genotypic p-value = 0.0002; allelic p-value = 0.004). This association was seen persistent with MTLE-HS (genotypic p-value = 0.0008; allelic p-value = 0.004) and also with JME (genotypic p-value = 0.01; allelic p-value = 0.05) cohort individually. In-silico functional prediction indicated that *ABCB1* rs1045642 has a deleterious impact on protein coding function and in splicing regulation. We conclude that the *ABCB1* and *ABCG2* variants do not confer to AED-resistance in the study population. However, *ABCB1* rs1045642 increases vulnerability to epilepsy with greater tendency for MTLE-HS in south Indian ancestry from Kerala.

## Introduction

Epilepsy constitutes a heterogeneous group of disorders characterized by recurrent unprovoked seizures due to widely different etiologies. It affects an estimated 50 million people worldwide, of which 80% reside in resource-poor countries [**1**]. Although majority of patients with epilepsy are responsive to presently available antiepileptic drugs (AEDs), nearly one-third of them continue to exhibit recurrent seizures despite optimal AED therapy [**2**]. Patients who are unresponsive to the first and second prescribed AEDs will often remain unresponsive to all other AEDs, including the newer ones, and to multiple AED combinations, indicating that they are multi-drug resistant from the beginning [**3**]. Role of efflux transporters in multidrug resistant epilepsy has been attributed from the studies which reported overexpression of the major blood–brain barrier transporter P-glycoprotein (P-gp) in epileptic tissue from patients with drug-resistant epilepsy [**4**]. This was further complemented by the over expression of other associated efflux proteins in the epileptogenic tissues both in human and animal models [**5**], [**6**], thus supporting the notion that efflux activity limits the AED availability to the targets.

The main efflux transporter proteins in the blood brain barrier constitute ATP-binding cassette (ABC) transporters super family of transmembrane proteins which efflux a wide range of substrates across cellular membranes at the expense of ATP hydrolysis. ABCB1 and ABCG2 are well characterized ABC transporters which showed marked cooperativity in efflux activity at the blood-brain barrier, evidenced from cellular localization and functional studies [**7**]. The P-gp/ABCB1, was the first identified ABC transporter superfamily for its role in contributing to AED resistance [**8**]. In vitro and in vivo studies have proved that substrate specificity of the AEDs to P-gp and intrinsic or acquired over expression of the P-gp can lead to extrusion of the drug, thereby limiting the intra parenchymal concentration of drugs. Such P-gp overexpression can be explained at least, in a part by the genetic variants in the *ABCB1* gene and might explain the clinical observation that patients with refractory epilepsy are usually resistant to a broad range of AEDs with different mechanisms of action [**9**].

Initial association of the *ABCB1* C3435T variant in multi-drug resistant epilepsy patients lead to the notion that the drug resistance in epilepsy might be genetically determined, where homozygous genotype of the C allele was more likely to be multidrug-resistant than the T allele [**10**]. This was further supported by the evidences from functional studies [**11**]–[**13**]. This association was subsequently replicated in a larger cohort along with the identification of associated haplotypes [**14**]. However, no other replication studies with the same definition, in other ethnicities, could reproduce the initial association, challenging the validity of the initial associations [**15**]–[**20**]. Although the efflux transporter, *ABCG2*, which codes for Breast cancer related protein (BCRP) contributes to integrity of blood brain barrier and has a considerable overlap in substrates with P-glycoprotein but pharmacogenetic data in epilepsy is scarce and inconsistent [**21**].

Pharmacogenetic studies in epilepsies till date have included all the epilepsy syndromes considering only their response profile, ignoring the underlying disease pathobiology, which can possibly have an effect in determining the AED response. These factors coupled with ethnic differences and variability in defining drug resistance among studies could significantly contribute for the inconsistencies in the results. Thus in the current study we have evaluated the association of *ABCB1* and *ABCG2* genetic polymorphisms with AED resistance in a uniform cohort of subjects with South Indian (Kerala) ancestry: MTLE-HS (prototype of AED-resistant epilepsy syndrome), JME (prototype of AED-responsive epilepsy syndrome) and ethnically matched non-epilepsy controls.

## Materials and Methods

### Study Cohorts

We recruited 738 ethnically matched Malayalam speaking subjects who were residents of Kerala, south India for more than three generations. Study subjects comprised of 259 patients with AED resistant MTLE-HS, 201 patients with AED responsive JME and 275 non-epilepsy control subjects. MTLE-HS was considered as a prototype of AED-resistant epilepsy while JME was considered as prototype of AED-responsive epilepsy as per the definition mentioned below. All the participants gave informed, written consent in a standard consent form to participate in the study after being provided with, and receiving a full explanation of study protocols and objectives. All potential participants who declined to participate or otherwise did not participate were eligible for treatment (if applicable) and were not disadvantaged in any other way by not participating in the study. The present study was approved by the Institutional Ethics Committee of Rajiv Gandhi Center for Biotechnology, India and Sree Chitra Tirunal Institute for Medical Sciences and Technology, India, established as per the Indian Council of Medical Research guidelines.

### AED-resistant Cohort

We defined AED-resistant patients as those who were unresponsive to at least two monotherapy and one duotherapy trials, each of ≥6 month duration and had seizure frequency ≥12 per year for ≥2 years. To conform to this definition, we recruited 259 MTLE subjects [**22**] who had pathologically verified HS after anterior temporal lobectomy (ATL), had no other lesions on magnetic response imaging and were seizure-free for at least one year following ATL. These patients had seizures for a mean duration of 18.7 (range 6 to 50) years, and had failed multiple AED trials prior to ATL. All of them had received at least two of the old AEDs (phenobarbital, phenytoin, carbamazepine and valproate), and half of them, in addition, had received at least one of the new AEDs (clobazam, lamotrigine, oxcarbazepine, topiramate and Levetiracetam) [**23**]. We have described our protocols for pre-surgical evaluation, selection for ATL and post-ATL follow-up in detail elsewhere [**24**], [**25**].

### AED-responsive Cohort

We defined AED-responsive patients as those who were free of seizures for ≥1 year on AED therapy. As a prototype of AED-responsive epilepsy, we recruited 201 patients with JME [**26**], who conformed to the following diagnostic criteria [**23**], [27], [**28**]: 1) bilateral myoclonic seizures involving the upper extremities, with age at onset between 8 and 25 years, occurring after awakening and without loss of consciousness with or without additional generalized tonic–clonic seizures and/or absence seizures; 2) otherwise normal neurological status and intelligence; and 3) normal background activity and paroxysmal generalized spike and wave discharges in the electroencephalogram (EEG). Abnormal EEG was utilized to support the diagnosis of JME, but was not mandatory for the diagnosis. The clinical and EEG characteristics of 183 of these JME patients have been published in detail elsewhere [**28**]. All the 201 JME patients fulfilled our criteria for AED-responsiveness, as defined above.

### Control Subjects

We randomly selected 275 subjects from the general population, who did not have personal or family history of epilepsy or any other neurological disorders as non-epilepsy healthy control cohort. Peripheral blood was collected from study subjects in EDTA-coated vials and DNA was isolated by the conventional phenol-chloroform method.

### SNP Selection, Genotyping and Statistical Analyses

In addition to the three well-studied polymorphisms in *ABCB1* viz: rs1128503 (C1236T), rs2032582 (G2677T) and rs1045642 (C3435T), in the present study we screened four additional variants, rs3213619 (T129C), rs2214102 (−1G/A), rs1202168 (+139C/T) and rs1922242 (−76T/A), which were selected based on the linkage disequilibrium status and potential functional importance. For *ABCG2* three functional variants viz: rs2231142 (Gln141Lys; missense), rs72552713 (Gln126Ter; stop gain) and rs2231137 (Val12Met; missense) were screened. Genotyping for *ABCB1* was performed by PCR-RFLP as per the conditions provided **(Table S1 in File S1)** and the selected SNPs in *ABCG2* was genotyped by fluorescence-based competitive allele-specific polymerase chain reaction (PCR) (KASPar) chemistry (KBiosciences, UK). The reaction comprised of 8 µl with 5 ng of DNA, 0.11 µl of assay mix and 4 µl of reaction mix and the PCR was performed in ABI 7500 real-time PCR System (Applied Biosystems, Foster City, CA, USA). The cycling conditions were as follows: 94°C for 15 min (Hot-start enzyme activation), 94°C for 20 s, a touchdown step for 10 cycles over 65–57°C for 60 s (dropping 0.8°C per cycle), and a final 26 step cycle with 94°C for 20 s and 57°C for 60 s. Further, the genotype calling based on the respective allele specific fluorescence was done by allelic discrimination utility of the SDS 7500 v2.0.5 software at an ambient temperature of 25°C and genotype clusters were plotted. PCR-RFLP genotyped SNPs were randomly selected and confirmed for concordance by sequencing (ABI PRISM Big Dye Terminator v3.1 cycle sequencing kit) according to the manufacturer’s instructions, and was analyzed using the ABI PRISM 3730 Genetic Analyzer (Applied Biosystems, Foster City, CA, USA).

Genotype and allele frequencies were computed and were checked for deviation from Hardy–Weinberg equilibrium. Fisher’s exact test (two-tailed) was used to compare allele frequencies between patients and control subjects. Statistical differences in genotype distributions were evaluated using Pearson’s χ2 test. Linkage disequilibrium (LD) pattern was plotted for the genotyped SNPs using Haploview 4.2 [**29**] and the haplotype analysis was performed using Unphased 3.1.5 [**30**] with 10,000 permutations for deriving empirical significance. We considered p-value of <0.05 as significant. Further GrASP V0.6 [**31**] was used to visualize, summarize and prioritize regions of interest from sliding window haplotype analysis, based jointly on the p-value from all the tests from these windows. We carried out Bonferroni’s correction to test for multiple comparisons.

Functional prediction of the deleterious effect if any, of the associated SNP of *ABCB1* and *ABCG2* with respect to the functional categories such as protein coding, splicing regulation, transcriptional regulation, and post-translation was assessed in-silico using F-SNP program (http://compbio.cs.queensu.ca/F-SNP/) [**32**]. F-SNP extracts information from large number of resources such as PolyPhen, SIFT, SNPeffect, SNPs3D, LS-SNP, Ensembl, ESEfinder, RescueESE, ESRSearch, PESX, TFSearch, Consite, GoldenPath, KinasePhos, OGPET, Sulfinator to generate a Functional Significance (FS) score. Further, a meta-analysis of the studies **(Table S2 in File S1)** that reported *ABCB1* C3435T allele frequencies in epilepsy patients and control subjects were performed to compare the prevalence of C3435T alleles (C vs T) among epilepsy patients and normal controls, using both fixed and random-effects models, using Review Manager 5.2 (http://review-manager.software.informer.com/5.2/). The inconsistency index I^2^ was used to assess between-study heterogeneity. A p-value of <0.05 was considered as significant throughout the analyses.

## Results

The demographic and clinical characteristics of the patients and controls are summarized in **Table 1**. We observed that all the studied variants in *ABCB1* and *ABCG2* were polymorphic and were within Hardy-Weinberg equilibrium (p>0.05) in healthy controls with exception to *ABCG2* variant rs72552713, which was found to be monomorphic and thus excluded from analysis. Further the samples which failed in genotype calling were also excluded from the analysis. We assessed the role of *ABCB1* and *ABCG2* variants in AED resistance by comparing a uniform cohort of AED resistant MTLE-HS epilepsy patients with AED responsive JME epilepsy patients. While for susceptibility to epilepsy we compared the epilepsy cohorts both individually and by pooling the cohorts against the healthy controls.

**Table 1:** Demographic and clinical characteristics of the patients and controls.

Subject Variables	MTLE/HS (n = 259)	JME (n = 201)	Controls (n = 278)
Male, n (%)	141(54.44)	104 (51.74)	147(52.87)
Female, n (%)	118(45.56)	97 (48.25)	131(47.12)
Age, mean ± SD	28.69±8.56	24.4±7.20	37.42±5.44
Age of first unprovoked sz (yrs.), mean ± SD	10.32±7.29	14.6±3.6	–
Duration of epilepsy, mean ± SD	18.58±9.37	15.1±5.2	–

MTLE-HS, mesial temporal lobe epilepsy/hippocampal sclerosis.

JME, Juvenile Myoclonic Epilepsy.

### *ABCB1* and *ABCG2* in AED Resistance

In the present study seven variants in *ABCB1* gene viz: rs3213619 (T129C), rs2214102 (−1G/A), rs1202168 (+139C/T), rs1128503 (C1236T), rs1922242 (−76T/A), rs2032582 (G2677T) and rs1045642 (C3435T) were screened for their contribution to the AED resistance. No significant differences in allelic and genotypic frequencies were observed, when the AED resistant group were compared with the AED responsive group **(** **Table 2** **)**. Subsequently we screened three functional variants in the *ABCG2* gene viz: rs2231142, rs72552713 and rs2231137 in the cohort of epilepsy patients. The genotypic and allelic frequencies of *ABCG2* variants did not differ significantly when the AED resistant patient group was compared with the AED responsive group **(** **Table 2** **)**. The results thus, rule out the role of *ABCB1* and *ABCG2* genetic variants in AED resistance.

**Table 2:** Genotype and allele frequency of *ABCB1 and ABCG2* SNPs in AED resistant and responsive cohorts.

SNP	Cohort	TT	TC	CC	*P*-value*	T	C	OR	95% C.I	*P*-value*
ABCB1	**AED RESISTANT**	240 (0.94)	15 (0.06)	0	0.61	495 (0.97)	15 (0.03)	1.209	0.58–2.54	0.70
rs3213619	**AED RESPONSIVE**	184 (0.93)	14 (0.07)	0		382 (0.965)	14 (0.035)			
		**GG**	**GA**	**AA**		**G**	**A**			
ABCB1	**AED RESISTANT**	247(0.965)	8 (0.031)	1 (0.004)	0.32	502 (0.98)	10 (0.02)	1.966	0.87–4.42	0.10
rs2214102	**AED RESPONSIVE**	186 (0.935)	11 (0.055)	2 (0.01)		383 (0.96)	15 (0.04)			
		**CC**	**CT**	**TT**		**C**	**T**			
ABCB1	**AED RESISTANT**	39(0.150	138 (0.53)	82 (0.32)	0.34	216 (0.42)	302 (0.58)	1.205	0.92–1.57	0.17
rs1202168	**AED RESPONSIVE**	25(0.125)	99 (0.495)	76 (0.38)		149 (0.37)	251 (0.63)			
		**CC**	**CT**	**TT**		**C**	**T**			
ABCB1	**AED RESISTANT**	32(0.12)	110(0.43)	117(0.45)	0.68	174 (0.34)	344 (0.66)	0.982	0.74–1.29	0.94
rs1128503	**AED RESPONSIVE**	29(0.145)	78(0.39)	93(0.465)		136 (0.34)	264 (0.66)			
		**TT**	**TA**	**AA**		**T**	**A**			
ABCB1	**AED RESISTANT**	152 (0.60)	98 (0.38)	6 (0.02)	0.98	402 (0.785)	110 (0.215)	1.029	0.75–1.41	0.87
rs1922242	**AED RESPONSIVE**	116 (0.59)	77 (0.39)	5 (0.02)		309 (0.78)	87 (0.22)			
		**GG**	**GT**	**TT**		**G**	**T**			
ABCB1	**AED RESISTANT**	29 (0.11)	129 (0.51)	98 (0.38)	0.28	187 (0.365)	325 (0.635)	1.015	0.77–1.33	0.94
rs2032582	**AED RESPONSIVE**	29 (0.15)	86 (0.43)	84 (0.42)		144 (0.36)	254 (0.64)			
		**CC**	**CT**	**TT**		**C**	**T**			
ABCB1	**AED RESISTANT**	12 (0.05)	136 (0.52)	111 (0.43)	0.70	160 (0.31)	358 (0.69)	1.106	0.84–1.46	0.52
rs1045642	**AED RESPONSIVE**	12 (0.06)	109 (0.54)	80 (0.40)		133 (0.33)	269 (0.67)			
		**AA**	**AG**	**GG**		**A**	**G**			
ABCG2	**AED RESISTANT**	6 (0.02)	54 (0.23)	179 (0.75)	0.27	66 (0.14)	412 (0.86)	0.926	0.63–1.35	0.70
rs2231137	**AED RESPONSIVE**	2 (0.01)	55 (0.275)	143 (0.715)		59 (0.15)	341 (0.85)			
		**CC**	**AC**	**AA**		**C**	**A**			
ABCG2	**AED RESISTANT**	190 (0.79)	45 (0.19)	4 (0.02)	0.82	425 (0.89)	53 (0.11)	0.901	0.58–1.39	0.66
rs2231142	**AED RESPONSIVE**	160 (0.81)	36 (0.18)	2 (0.01)		356 (0.90)	40 (0.10)			

OR, Odds ratio; CI, Confidence Interval.

*uncorrected p-value.

### *ABCB1* and *ABCG2* in Susceptibility to Epilepsy

When *ABCB1* variants were screened for their contribution to susceptibility to epilepsy by comparing epilepsy cohorts (MTLE-HS and JME) against the healthy controls, we observed significant association with rs1045642 (C3435T) at genotypic (p = 0.0008, p_corrected = _0.005) and allelic level (p = 0.004, p_corrected_ = 0.02, OR = 1.44, 95% C.I = 1.12–1.85) with an over representation of T allele and TT genotype in MTLE-HS. This association was also observed with JME at genotypic level (p = 0.01, p_corrected_ = 0.07) and allelic levels (p = 0.05, p_corrected_ = 0.35, OR = 1.3, 95% CI = 0.99–1.70). However, in the JME cohort association did not survive Bonferonni’s correction for multiple testing. The significance persisted both at genotypic (p = 0.0002, p_corrected = _0.001) and allelic (p = 0.004, p_corrected = _0.02, OR = 1.38, 95% CI = 1.10–1.71) levels when the epilepsy samples were pooled and compared with the normal healthy control **(** **Table 3** **)**. Further, using an additive model for association analysis (TT+CT vs CC) we confirm that the association is influenced by T allele in genotypic combination with AED resistant group (p = 0.0002, OR = 3.35, 95% CI = 1.71–6.57), AED responsive group (p = 0.004, OR = 2.57, 95% CI = 1.3–5.04) and epilepsy samples pooled together (p = 0.00003, OR = 2.96, 95% CI = 1.74–5.04) when compared with healthy controls respectively **(Table S3 in File S1)**. The results thus show the T allele has an increased vulnerability to develop epilepsy. The study also indicates that the T allele has a higher tendency to influence the MTLE-HS than the JME.

**Table 3:** Genetic association of the *ABCB1* rs1045642 with epilepsy.

Cohort	CC	CT	TT	*P*-value*	C	T	OR	C.I	*P*-value*
MTLE-HS	12(0.05)	136(0.52)	111(0.43)	**0.0008**	160(0.31)	358(0.69)	1.44	1.121 to 1.858	**0.004**
NORMAL CONTROLS	39(0.14)	140(0.50)	99(0.36)		218(0.39)	338(0.61)			
JME	12(0.06)	109(0.54)	80(0.40)	**0.01**	133(0.33)	269(0.67)	1.30	0.997 to 1.707	**0.05**
NORMAL CONTROLS	39(0.14)	140(0.50)	99(0.36)		218(0.39)	338(0.61)			
MTLE-HS+JME	24(0.05)	245(0.53)	191(0.42)	**0.0002**	293(0.32)	627(0.68)	1.38	1.108 to 1.719	**0.004**
NORMAL CONTROL	39(0.14)	140(0.50)	99(0.36)		218(0.39)	338(0.61)			

OR, Odds ratio; CI, Confidence Interval.

*uncorrected p-value.

**Figure 1:**
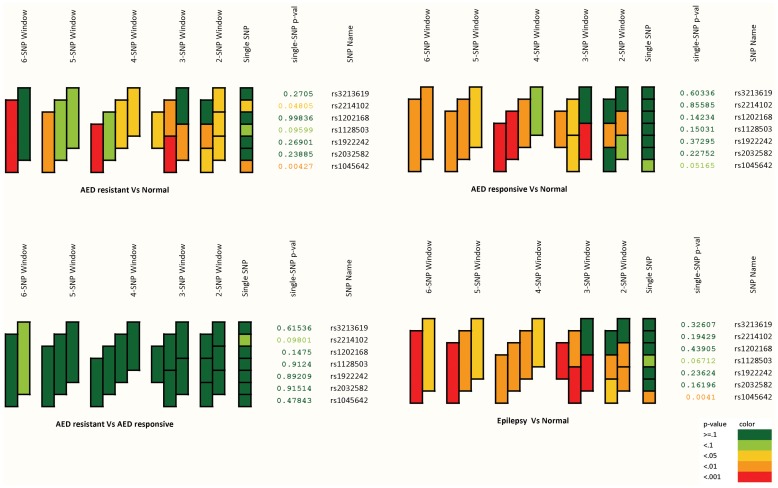
The haplotype visualization based on the p-values from sliding window haplotype tests.

The haplotype visualization based on the p-values from sliding window haplotype tests is shown in **Table 4** (**Figure 1** **)** depicting the T allelic association extending in haplotypic combination with epilepsy. While comparing the haplotypic association among the three well studied ABCB1 variants viz: rs1128503 (C1236T), rs2032582 (G2677T) and rs1045642 (C3435T) we observed that the TGT haplotype was strongly associated with epilepsy but not with AED resistance **(** **Table 4** **)**. The alternate haplotype acts as a protective against epilepsy. In-silico analysis of these SNP using F-SNP program indicated that rs1045642 (C3435T) has a significant role in deleterious impact on protein coding function and in splicing regulation with FS Score of 0.528 **(** **Table 5** **)**. Interestingly, in-silico prediction of the SNPs involved in haplotypic combinations such as rs1128503 (FS score 0.407), rs2032582 (FS score 0.418) and rs1045642 (FS score 0.528) are also likely to have an add-on functional effect in protein coding function, splicing regulation and post-translational changes **(** **Table 5** **)**. No significant differences in allele or genotypes of *ABCG2* variants were found between healthy controls and the epilepsy (MTLE-HS, JME) cohorts. The results obtained from the *ABCG2* ruled out the role of *ABCG2* polymorphisms in determining predisposition to epilepsy.

**Table 4:** Three locus haplotypes (Risk and protective) of *ABCB1* variants C1236T, G2677T and C3435T.

Effect	Cohort	rs1128503 (C1236T)	rs2032582 (G2677T)	rs1045642 (C3435T)	Case frequency	Control frequency	OR	95% CI	*P*-value
	MTLE-HS vs Healthy Control	T	G	T	0.039	0.012	4.68	1.70–12.87	0.007
Risk	JME vs Healthy Control	T	G	T	0.030	0.012	3.89	1.30–11.57	0.04
	MTLE-HS+JME vs Healthy Control	T	G	T	0.035	0.012	4.34	1.66–11.33	0.009
	MTLE-HS vs Healthy Control	C	G	C	0.194	0.279	1.56	1.14–2.13	0.001
Protective	JME vs Healthy Control	C	G	C	0.183	0.279	1.58	2.22–1.12	0.001
	MTLE-HS+JME vs Healthy Control	C	G	C	0.189	0.279	1.57	1.19–2.05	0.0001

OR, Odds ratio; CI, Confidence Interval.

**Table 5:** In-silico functional prediction of critical variants of *ABCB1* SNPs.

SNP	FunctionalCategory	Prediction tool	Prediction effect	FS score
rs1128503	Splicingregulation	ESEfinder	Not changed	0.407
		ESRSearch	Changed	
		PESX	Changed	
		RESCUE ESE	Changed	
rs2032582	Proteincoding	PolyPhen	Benign	0.418
		SIFT	No prediction	
		SNPeffect	Deleterious	
		LS-SNP	Deleterious	
		SNPs3D	Deleterious	
		Ensembl-NS	Nonsynonymous	
	Splicingregulation	ESEfinder	Changed	
		ESRSearch	Changed	
		PESX	Not changed	
		RESCUE ESE	Not changed	
	Posttranslation	KinasePhos	Not exist	
		OGPET	Exist	
		Sulfinator	Not exist	
rs1045642	Proteincoding	PolyPhen	Benign	0.528
		SIFT	Tolerated	
		SNPeffect	Benign	
		LS-SNP	Benign	
		SNPs3D	Deleterious	
		Ensembl-NS	Nonsynonymous	
	Splicingregulation	ESEfinder	Changed	
		ESRSearch	Changed	
		PESX	Changed	
		RESCUE ESE	Not changed	
	Posttranslation	KinasePhos	Not exist	
		OGPET	Not exist	

FS Score; Functional significance score.

F-SNP: http://compbio.cs.queensu.ca/F-SNP/.

**Figure 2:**
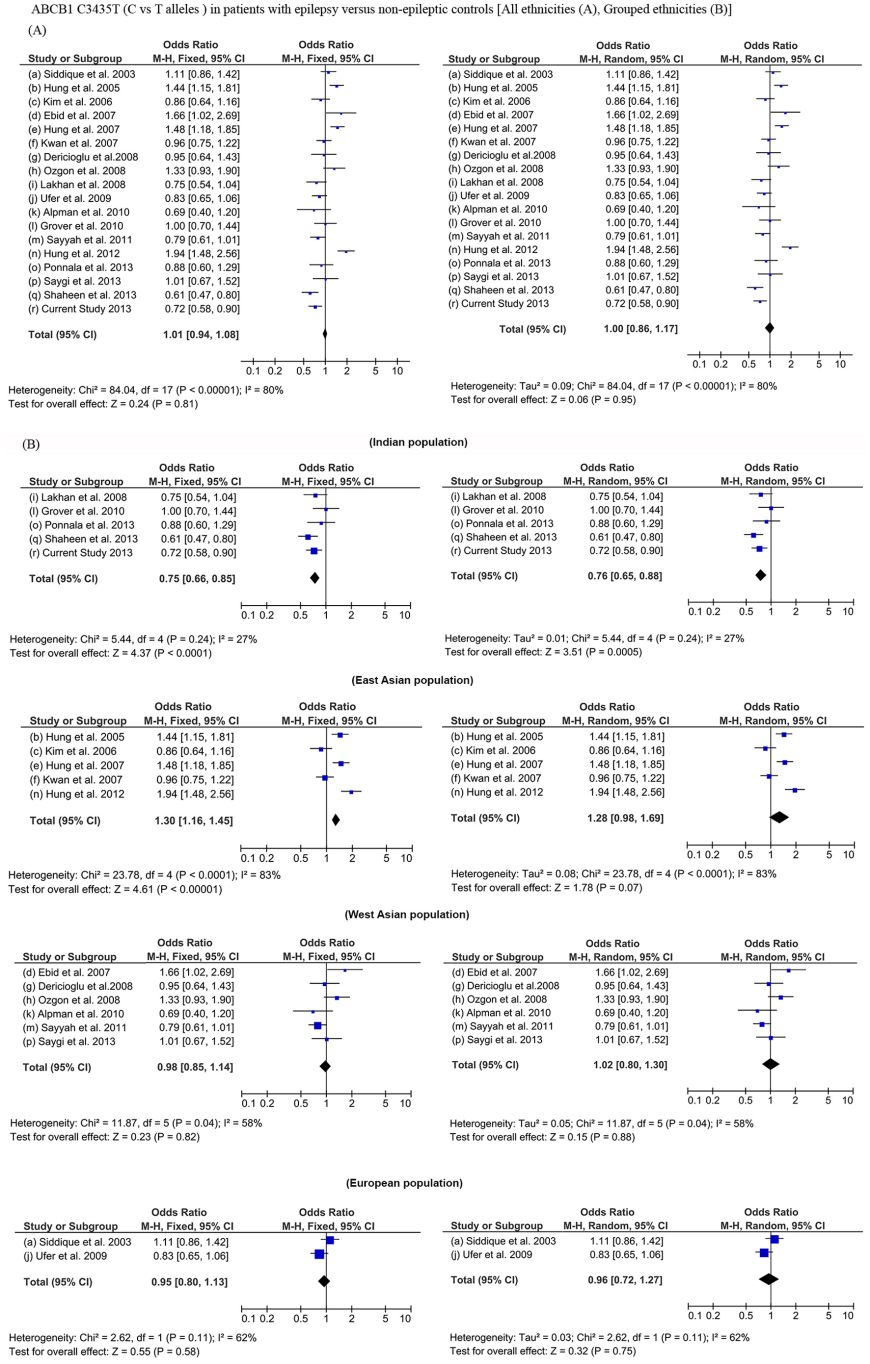
Meta-analysis of *ABCB1* C3435T (C vs T alleles) in patients with epilepsy and non-epileptic controls across all ethnicities (A) and grouped ethnicities (B).

We also performed a meta-analysis of 17 previously reported studies along with the current study to compare the prevalence of C3435T alleles (C vs T) among epilepsy patients and normal controls, using both fixed and random-effects models **(Table S2 in File S1)**. Meta-analysis could not predict any significant association of C3435T with epilepsy susceptibility across various ethnicities **(** **Figure 2A** **)**. A high heterogeneity among the studies was observed with high I^2^ values. Interestingly, when we performed a meta-analysis by stratifying these 18 studies based on specific ethnicities such as Indian, East asian, European and west Asian we observed that Indian ethnicity show a significant association with T allele in both fixed effect and random effect model with epilepsy **(** **Figure 2B** **)**. Studies in the East Asian population showed an association with C allele (fixed effect); however, the heterogeneity was higher among the studies.

## Discussion

Pharmacogenetic studies in epilepsy involving the AED efficacy have mainly focused on the genes involved in the AED pharmacokinetics, especially the multi-drug transporters. Increased functionality of the efflux transporters in drug-resistant epileptic conditions made them the excellent candidate genes to query for the genetic association of AED-resistance. After the influential report of the association of rs1045642 (C3435T) in *ABCB1* with AED-resistance [**10**], a series of studies were reported, further extending to other variants viz: rs1128503 (C1236T), rs2032582 (G2677T) in the gene individually and in haplotype combinations, supporting and negating the initial association [**33**]–[**35**]. The present study investigated the role of allelic variants in ATP-binding cassette protein family of genes viz: *ABCB1* and *ABCG2,* in predicting the AED-resistance in uniform well-characterized cohorts of AED-resistant MTLE-HS patients and AED-responsive JME patients from the south Indian population of Kerala. Further, the study also inquired the role of *ABCB1* and *ABCG2* in susceptibility to epilepsy by comparing the epilepsy cohorts with non-epilepsy controls. In line with the results from previous studies, no significant differences in allele and genotypic frequencies of *ABCB1* and *ABCG2* variants were observed between MTLE-HS and JME cohorts, thereby, ruling out the role of *ABCB1* and *ABCG2* variants in determining the AED-resistance in epilepsy. Unlike a majority of previous studies, we explicitly defined AED-responsive and AED-resistant cohorts. Interestingly, we observed that *ABCB1* variants have a role in predisposing to epilepsy.

Studies which enquired genetic determinants of *ABCB1* associated with AED-resistance have produced discrepant results which can be accounted for a variety of factors such as phenotype definition of the AED-resistance, ethnicity and differences in substrate specificity of AEDs to P-gp. In general, AED-resistance was defined as seizure recurrence despite the trial of two to three AEDs or surgical intervention for seizure control, and the AED-responsiveness as seizure-freedom on AEDs at a specific period of follow-up [**36**] **.** Interestingly, all the epilepsy pharmacogenetic studies were different in majority of these parameters. The minimum period of follow-up required to define AED-resistance were found to be most variable among the studies, which ranged from 6 months to 2 years, and the seizure frequency during the period of follow-up ranged from one seizure per month to less than one per year or seizure-free at last follow-up [**37**], thereby making the patient groups defined as drug-resistant in one study as drug-responsive in another**.** Furthermore, cohorts selected for pharmacogenetic studies have often comprised of heterogeneous groups of epilepsy syndromes with widely differing AED-responsiveness and underlying disease pathobiology [**38**]. To reduce the phenotypic disparity and thereby to minimize the underlying genetic heterogeneity, we included patients who had failed multiple AEDs, had undergone resective surgery and had verified HS to comprise the AED-resistant cohort, and clinically well-characterized JME patients to represent the AED-responsive cohort. Our definition of the AED-resistance was also in line with the recent ILAE proposal [**39**].

Population stratification could be another significant component in having discrepant observation which could be mediated by issues of genetic heterogeneity within and between the populations. It is known that ethnically different population exhibit marked variation in the allele frequency distribution of SNPs and also linkage disequilibrium patterns. To avoid genetic heterogeneity among study subjects we selected Malayalam-speaking genetically homogenous Dravidian populations of Kerala, which has been reported to be genetically distinct from north Indian and other world populations [**40**]. This ethno-geographical differences in allele frequency distribution is further evident where C allele and CC genotype was associated with drug resistance in Caucasians [**10**] while alternate TT genotype has been implicated in drug resistance in Japanese and Chinese population [**41**], [**42**]. Even within the Indian subjects the distribution of the T allele was observed to be higher in the south Indian drug resistant epilepsy patients when compared to its counterpart in north Indian population [**43**], while frequencies of AED responsive patients were comparable among both populations **(Figure S1)**. The current study also could not observe any strong pair-wise LD in the studied SNP loci and among the three locus rs1128503 (C1236T), rs2032582 (G2677T) and rs1045642 (C3435T) in Kerala population **(Figure S2)**, which was in contrast to north Indian population [**44**] and also to Taiwanese and Japanese epilepsy patient population [**42**], [**45**]. Ethno-geographical differences in allele frequency distribution was also reflected in the haplotype distributions of these three loci, where it was found to be differing in AED resistant and responsive epilepsy cohort among different ethnicities studied **(Table S4 in File S1)**. This increased levels of diversity between populations could be indicative of positive selection, resulting in the allele frequency changes in the population under selection pressure, which may be mediated by distinct environmental or cultural influences as has been suggested earlier [**46**], [**47**].

Even with a strict phenotypic definition and a well stratified ethnic population we could not identify any significant role of *ABCB1* and *ABCG2* variants with AED resistance. Interestingly the variant rs1045642 (C3435T) indicated a significant association with an over representation of TT genotype and T allele in epilepsy patients, when the AED resistant cohort and AED responsive cohort were compared independently with the normal healthy controls. This significance persisted when both the cohorts were pooled and compared with the normal healthy controls indicating its role in susceptibility to epilepsy. An association between the ABCB1 gene and the development of epilepsy has been reported with an overrepresentation of C allele in patient group [**45**], [**48**]. A meta-analysis performed earlier using allele frequencies from nine studies, ruled out an association between ABCB1 C3435T polymorphisms and the risk of developing epilepsy [**49**]. Subsequent to this meta-analysis several studies have been added and a recently published study from South Indian epilepsy patients also reported ABCB1 C3435T association with T allele overrepresentation showing ethnicity specific association [**50**]. We performed a meta-analysis by incorporating 18 studies including the present study representing global ethnicity we observe no statistical significance of the ABCB1 C3435T alleles in epilepsy. This could be accounted to the heterogeneity among the studies as a result of the allele frequency distribution in different ethnicities and the disparities in clinical phenotypes of epilepsy between the studies. However, a significant association with T allele in both fixed effect and random effect model was observed when meta-analysis was confined to Indian studies and this also minimized the study heterogeneity. In a fixed effect model East Asian ethnicity predicted an association with C allele while the study heterogeneity was observed to be high. These evidences indicate the the of *ABCB1* variant with epilepsy susceptibility was ethnicity specific.

Although, a significant association of *ABCB1* C3435T variant was observed biological significance of the association still seems to be elusive. In-silico prediction of functional implication of rs1045642, do suggest that this SNP in isolation or in haplotypic combination with rs1128503 and rs2032582 can have deleterious impact on protein coding function, splicing regulation and post-translational changes. Recent studies in animal models of repetitive seizures have shown the role of P-gp in contributing to hippocampal and neocortical cell membrane depolarization, thus suggestive of a possible contribution in epileptogenesis [**51**]. Furthermore, it was also shown that the variant rs1045642 influences normal patterns of event-related potential generation during auditory target detection and novelty processing [**52**] and also influences the amplitude of auditory P300, event-related potential in schizophrenia patients [**53**]. Studies have also reported association of the *ABCB1* variant rs1045642 (C3435T) with major depressive disorder in Japanese [**54**]. These evidences do indicate the potentiality of *ABCB1* in increasing the risk of epilepsy but however, the exact mechanism still remains elusive and warrants further studies.

Our results also confronts the substrate specificity of P-gp for AEDs on which the transporter hypothesis was built [**38**]. Various *in vitro* and *in vivo* studies suggests that all AED are not substrates for P-gp and the degree of affinity to different AEDs varies, again with conflicting findings from different studies [**55**], thus compelling the need of studies that include AEDs specifically transported by P-gp. However, in a clinical scenario, designing such a study seems improbable as the objective of pharmacotherapy of epilepsy is to achieve seizure-freedom with available and affordable AEDs. While addressing the transporter hypothesis our study also could not show a role of *ABCG2* variants with AED-resistance in epilepsy, and similar lack of association was also observed with from non-ABC transporters, such as major vault protein [**56**]. Patients whose seizures are poorly responsive would have received several trials with multiple AEDs, often in varying combinations by the time they present to tertiary referral centers. Many of these AEDs have multiple targets. Therefore functional significance of drug target genes and drug metabolizing genes should also be investigated in associating the role of AEDs with therapeutic response. Pharmacogenetic studies in epilepsies till date have included all the epilepsy syndromes considering only their response profile, ignoring the underlying disease pathobiology, which can possibly have an effect in determining the AED response. Transporter hypothesis has been the most preferred choice for understanding the drug resistance and often ignoring the likelihood of a multifactorial nature of drug resistance and the complexity of the events regulating transporters and the role of genetic variants in drug targets. In an earlier study on the same population we reported that a synonymous variant in *GABRG2* rs211037 and *SCN1A* rs3812718 may have a putative role in increasing the risk of epilepsy, which was independent of its phenotype, that is, MTLE-HS or Juvenile myoclonic epilepsy [**23**], [**57**]. Functionally significant association of drug target genes such as *GABRG2* and *SCN1A* and efflux transporter gene *ABCB1* to susceptibility to epilepsy in the present population needs to be seen in combination rather than isolation in evaluating therapeutic response. In a recent study on single drug therapy, it has been reported that genetic variants in *SCN1A*, *EPHX1* and *UGT2B7* genes interactively affect the concentration–dose ratio of carbamazepine [**58**]. We conclude that variants in the *ABCB1*and *ABCG2* do not confer a significant risk to AED-resistance in South Indian population of Kerala. but instead increases vulnerability to epilepsy and associated phenotypes. We further suggest that combinatorial effects of genetic variants should be utilized in understanding the phenotypic variants in epilepsy and only then the pharmacogenetic traits will provide meaningful observations in defining and evaluating drug resistance in epilepsies.

## Supporting Information

Figure S1 **Comparison of C3435T allele frequency in epilepsy patients from Indian studies.**
(TIF)

Figure S2 **Linkage disequilibrium Plot of** ***ABCB1*** **variants in the AED-resistant MTLE-HS patients, AED-responsive JME patients and normal controls from the south Indian population of Kerala.**
(TIF)

File S1 **Tables S1–S4.** Table S1: Primer sequences, PCR conditions, Restriction enzymes and RFLP fragment sizes of the genotyped variants in *ABCB1. ABCG2*. Kaspar genotyping was done as per the manufacturer’s protocol. Table S2: Studies included in the meta-analysis to compare the prevalence of *ABCB1* C3435T alleles (C vs T) among epilepsy patients and normal controls. Table S3: Model based genetic association of the ABCB1 rs1045642 with Epilepsy. Table S4: Comparison of haplotype frequency of *ABCB1* three locus haplotypes (C1236T, G2677T and C3435T) in epilepsy patients across studies.
(DOC)
