# Supplementary figures and images for: Genetic Association Analysis of ATP Binding Cassette Protein Family Reveals a Novel Association of ABCB1 Genetic Variants with Epilepsy Risk, but Not with Drug-Resistance
Source: PLoS One. 2014 Feb 21;9(2):e89253. doi: 10.1371/journal.pone.0089253 (PMC3931716; doi:10.1371/journal.pone.0089253)

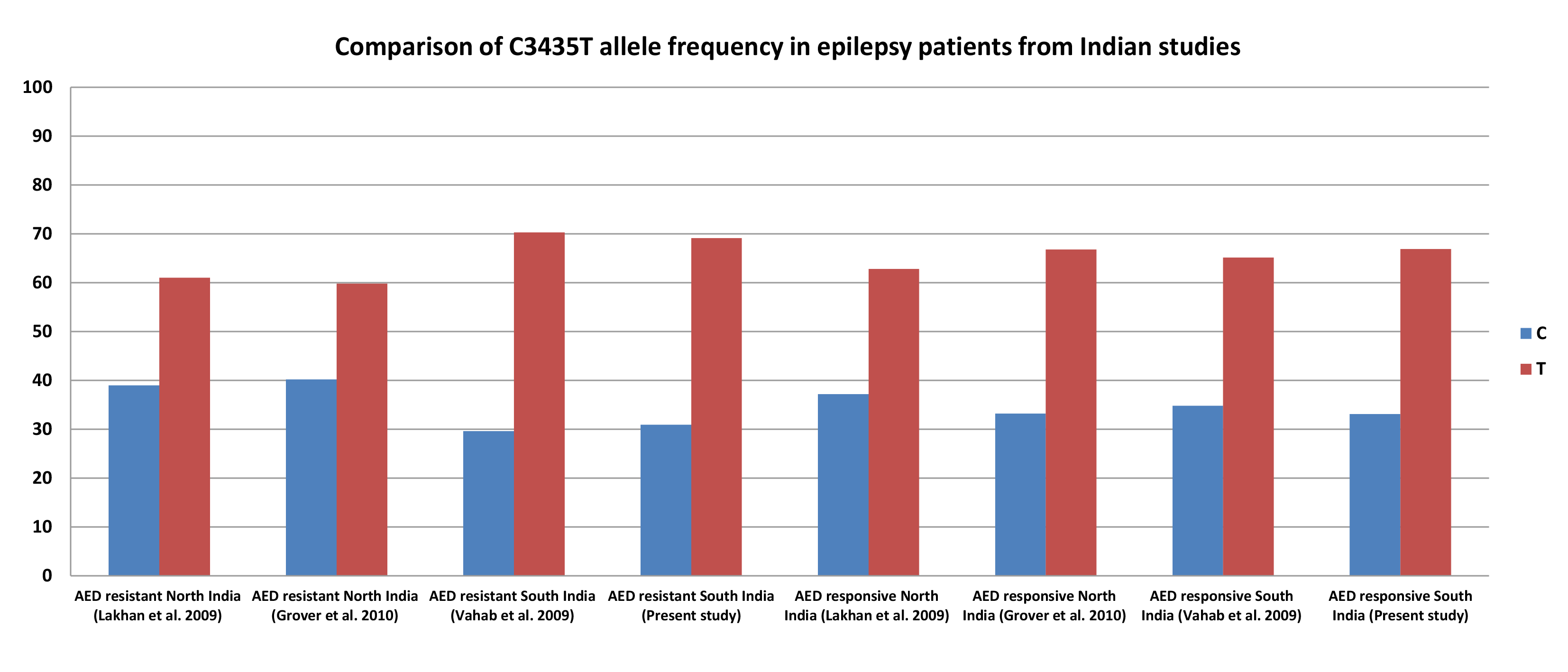

Supplement: Figure S1 — Comparison of C3435T allele frequency in epilepsy patients from Indian studies. (TIF) [file pone.0089253.s001.tif]

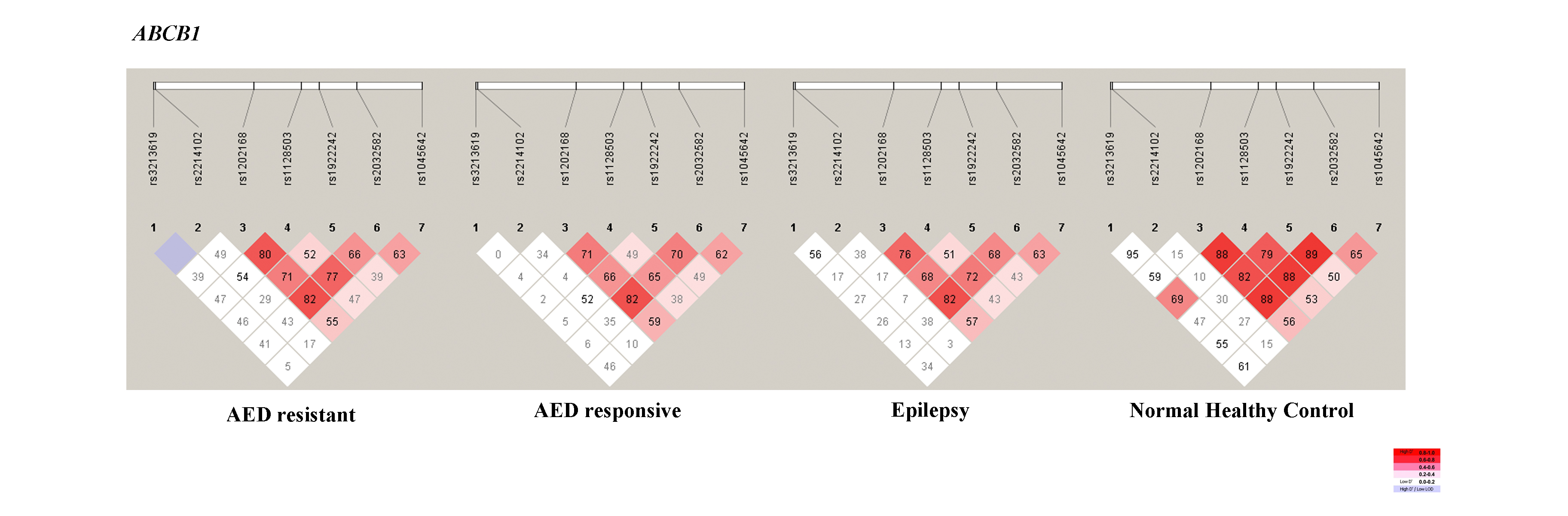

Supplement: Figure S2 — Linkage disequilibrium Plot of ABCB1 variants in the AED-resistant MTLE-HS patients, AED-responsive JME patients and normal controls from the south Indian population of Kerala. (TIF) [file pone.0089253.s002.tif]
