# Supplementary material for: Genetic Association Analysis of ATP Binding Cassette Protein Family Reveals a Novel Association of ABCB1 Genetic Variants with Epilepsy Risk, but Not with Drug-Resistance
Source: PLoS One. 2014 Feb 21;9(2):e89253. doi: 10.1371/journal.pone.0089253 (PMC3931716; doi:10.1371/journal.pone.0089253)
Supplement: File S1 — Tables S1–S4. Table S1: Primer sequences, PCR conditions, Restriction enzymes and RFLP fragment sizes of the genotyped variants in ABCB1. ABCG2. Kaspar genotyping was done as per the manufacturer’s protocol. Table S2: Studies included in the meta-analysis to compare the prevalence of ABCB1 C3435T alleles (C vs T) among epilepsy patients and normal controls. Table S3: Model based genetic association of the ABCB1 rs1045642 with Epilepsy. Table S4: Comparison of haplotype frequency of ABCB1 three locus haplotypes (C1236T, G2677T and C3435T) in epilepsy patients across studies. (DOC) [file pone.0089253.s003.doc]

**Table S1**: Primer sequences, PCR conditions, Restriction enzymes and RFLP fragment sizes of the genotyped variants in *ABCB1. ABCG2*. Kaspar genotyping was done as per the manufacturer’s protocol.

| **SNP** | **Primer Sequence**  **5🡪 3** | **Annealing temperature** | **Restriction enzymes** | **Homozygous** | **Heterozygous** |
| --- | --- | --- | --- | --- | --- |
| ***ABCB1*** |  |  |  |  |  |
| rs3213619 | F- TCAGCATTCAGTCAATCCGG  R- TTTGCGTGCCCCTACCTC | 62.5^0^C | MspA1I | TT 170, 103  CC 138, 103, 32 | CT 170, 138, 103, 32 |
| rs2214102 | F-TCTTACTGCTCTCTGGCTTCG  R-CATTTATTTCAGAGCTGGAGGC | 62.1^0^C | FokI | AA 238  GG 119 | GA 238, 119 |
| rs1202168 | F-AGGTTTCATTTTGGTGCCTG  R-GAACAAAAGGATGCACACGAC | 56.3^o^C | SspI | CC 299  TT 275, 24 | CT 299, 275, 24 |
| rs1128503 | F-TATCCTGTGTCTGTGAATTGCC  R-CCTGACTCACCACACCAATG | 52.3^o^C | HaeIII | CC 269,62,35  TT 269,97 | CT 269, 97, 62, 35 |
| rs1922242 | F-TTTGTCAACATTTTTTTGAAGC  R- TATTATTGCAAATGCTGGTTGC | 54.5^o^C | ApoI | TT 231,84  AA 120, 111, 84 | TA 231, 120, 111, 84 |
| rs2032582 | F-TGCAGGCTATAGGTTCCAGG  R- TTTAGTTTGACTCACCTTCCCG | 53.2^o^C | BanI | TT 224  GG 198, 26 | GT 224, 198, 26 |
| rs1045642 | F-TGTTTTCAGCTGCTTGATGG  R-AAGGCATGTATGTTGGCCTC | 61.3^o^C | DpnII | TT 197  CC 158, 39 | CT 197, 158, 39 |
| ***ABCG2*** |  |  |  |  |  |
| rs2231142 | KASPar genotyping assay | 57^o^C |  |  |  |
| rs72552713 | KASPar genotyping assay | 57^o^C |  |  |  |
| rs2231137 | KASPar genotyping assay | 57^o^C |  |  |  |

**Table S2**: Studies included in the meta-analysis to compare the prevalence of *ABCB1* C3435T alleles (C vs T) among epilepsy patients and normal controls

| **Study** | | **Ethnicity** | **Cohort** | **(n)** | **C allele** | **T allele** |
| --- | --- | --- | --- | --- | --- | --- |
| 1 | Siddique et al. 2003 | UK | Epilepsy | 315 | 315 | 315 |
|  |  |  | Normal Control | 200 | 190 | 210 |
| 2 | Hung et al. 2005 | Taiwan | Epilepsy | 331 | 320 | 342 |
|  |  |  | Normal Control | 287 | 226 | 348 |
| 3 | Kim et al. 2006 | Korea | Epilepsy | 160 | 191 | 129 |
|  |  |  | Normal Control | 212 | 268 | 156 |
| 4 | Ebid et al. 2007 | Egypt | Epilepsy | 100 | 121 | 79 |
|  |  |  | Normal Control | 50 | 48 | 52 |
| 5 | Hung et al. 2007 | Taiwan | Epilepsy | 327 | 320 | 334 |
|  |  |  | Normal Control | 287 | 226 | 348 |
| 6 | Kwan et al. 2007 | China | Epilepsy | 746 | 935 | 557 |
|  |  |  | Normal Control | 179 | 228 | 130 |
| 7 | Dericioglu et al. 2008 | Turkey | Epilepsy | 89 | 86 | 92 |
|  |  |  | Normal Control | 100 | 99 | 101 |
| 8 | Ozgon et al. 2008 | Turkey | Epilepsy | 97 | 113 | 81 |
|  |  |  | Normal Control | 174 | 178 | 170 |
| 9 | Lakhan et al. 2008 | India | Epilepsy | 325 | 250 | 400 |
|  |  |  | Normal Control | 97 | 88 | 106 |
| 10 | Ufer et al. 2009 | Germany | Epilepsy | 291 | 259 | 323 |
|  |  |  | Normal Control | 242 | 238 | 246 |
| 11 | Alpman et al. 2010 | Turkey | Epilepsy | 38 | 32 | 44 |
|  |  |  | Normal Control | 87 | 89 | 85 |
| 12 | Grover et al. 2010 | India | Epilepsy | 212 | 153 | 271 |
|  |  |  | Normal Control | 93 | 67 | 119 |
| 13 | Sayyah et al. 2011 | Iran | Epilepsy | 332 | 267 | 397 |
|  |  |  | Normal Control | 200 | 184 | 216 |
| 14 | Hung et al. 2012 | China | Epilepsy | 234 | 280 | 188 |
|  |  |  | Normal Control | 189 | 164 | 214 |
| 15 | Ponnala et al. 2013 | India | Epilepsy | 127 | 94 | 160 |
|  |  |  | Normal Control | 100 | 80 | 120 |
| 16 | Saygi et al. 2013 | Turkey | Epilepsy | 119 | 118 | 120 |
|  |  |  | Normal Control | 74 | 73 | 75 |
| 17 | Shaheen et al. 2013 | India | Epilepsy | 220 | 176 | 264 |
|  |  |  | Normal Control | 220 | 229 | 211 |
| 18 | Current study 2013 | India | Epilepsy | 460 | 293 | 627 |
|  |  |  | Normal Control | 278 | 218 | 338 |

Note. The meta-analysis included published studies till 2013 which have reported *ABCB1* C3435T allele frequencies in epilepsy patients and control subjects. The epilepsy cohort included all epilepsy patients pooled from individual studies irrespective of AED resistant or responsive, thus totaling 4523 epilepsy cases and 3069 controls.

**Table S3**: Model based genetic association of the ABCB1 rs1045642 with Epilepsy

| **Cohort** | **CC+CT** | **TT** | **OR** | **C.I** | ***P* *** | **TT+CT** | **CC** | **OR** | **C.I** | ***P**** |
| --- | --- | --- | --- | --- | --- | --- | --- | --- | --- | --- |
| **MTLE-HS** | 148(0.57) | 111(0.43) | 1.13 | 0.78 - 1.65 | 0.57 | 247(0.95) | 12(0.05) | 1.31 | 0.57 - 2.97 | 0.5337 |
| **JME** | 121(0.60) | 80(0.40) |  |  |  | 189(0.94) | 12(0.06) |  |  |  |
| **MTLE-HS** | 148(0.57) | 111(0.43) | 1.36 | 0.96 - 1.92 | 0.09 | 247(0.95) | 12(0.05) | 3.36 | 1.72 - 6.57 | **0.0002** |
| **NORMAL CONTROL** | 179(0.64) | 99(0.36) |  |  |  | 239(0.86) | 39(0.14) |  |  |  |
| **JME** | 121(0.60) | 80(0.40) | 1.19 | 0.82 - 1.74 | 0.39 | 189(0.94) | 12(0.06) | 2.57 | 1.31 - 5.05 | **0.0044** |
| **NORMAL CONTROL** | 179(0.64) | 99(0.36) |  |  |  | 239(0.86) | 39(0.14) |  |  |  |
| **MTLE-HS + JME** | 269(0.58) | 191(0.42) | 1.28 | 0.94 - 1.75 | 0.12 | 436(0.95) | 24(0.5) | 2.96 | 1.74 - 5.05 | **0.00003** |
| **NORMAL CONTROL** | 179(0.64) | 99(0.36) |  |  |  | 239(0.86) | 39(0.14) |  |  |  |

OR, Odds ratio; CI, Confidence Interval

*uncorrected p-value

**Table S4**: Comparison of haplotype frequency of *ABCB1* three locus haplotypes (C1236T, G2677T and C3435T) in epilepsy patients across studies

| **Haplotype** | **Zimprich et al. 2004** | | **Hung et al. 2005** | | **Kim et al. 2006** | | **Seo et al. 2006** | | **Vahab et al. 2009** | | **Lakhan et al. 2009** | |
| --- | --- | --- | --- | --- | --- | --- | --- | --- | --- | --- | --- | --- |
|  | **AED resistant-Frequency (%)** | **AED responsive -Frequency (%)** | **AED resistant-Frequency (%)** | **AED responsive -Frequency (%)** | **AED resistant-Frequency (%)** | **AED responsive -Frequency (%)** | **AED resistant-Frequency (%)** | **AED responsive -Frequency (%)** | **AED resistant-Frequency (%)** | **AED responsive -Frequency (%)** | **AED resistant-Frequency (%)** | **AED responsive -Frequency (%)** |
| **CGC** | 45.6 | 46.9 | 4 | 35.2 | 17.24 | 19.91 | 19.6 | 15.1 | 13.6 | 8.2 | 19.3 | 22.9 |
| **TTT** | 39.5 | 39.5 | 6.9 | 30.6 | 15.74 | 17.12 | 30.4 | 42.1 | 25.2 | 25.6 | 40.9 | 44.7 |
| **CGT** | 9.9 | 8.3 | 28.5 | 1.4 | - | - | 3 | 4 | 9.9 | 7.9 | 4.8 | 4.3 |
| **TTC** | 2.2 | 2.1 | 34.1 | 5.1 | 9.13 | 10.4 | 10.7 | 6.7 | 6.2 | 2.4 | 7.4 | 5.9 |
| **CTT** | 1.5 | 1.2 | 1.6 | 0.9 | 1.44 | 0.79 | 1.8 | 0.8 | 22.8 | 26.9 | 5.4 | 7.4 |
| **Others** | 1.3 | 1.9 | 24.9 | 26.8 | 56.45 | 51.79 | 34.5 | 31.4 | 22.3 | 29 | 22.2 | 14.9 |
| **Haplotype** | **Grover et al. 2010** | | **Haerian et al. 2011 (Chinese)** | | **Haerian et al. 2011 (Indian)** | | **Haerian et al. 2011 (Malay)** | | **Haerian et al. 2011 (Combined)** | | **Kerala population (present study)** | |
|  | **AED resistant-Frequency (%)** | **AED responsive -Frequency (%)** | **AED resistant-Frequency (%)** | **AED responsive -Frequency (%)** | **AED resistant-Frequency (%)** | **AED responsive -Frequency (%)** | **AED resistant-Frequency (%)** | **AED responsive -Frequency (%)** | **AED resistant-Frequency (%)** | **AED responsive -Frequency (%)** | **AED resistant-Frequency (%)** | **AED responsive -Frequency (%)** |
| **CGC** | 22.2 | 29.5 | 18 | 20 | 16 | 11 | 24 | 15 | 19 | 18 | 19.4 | 18.3 |
| **TTT** | 52.6 | 51.6 | 28 | 14 | 23 | 25 | 21 | 20 | 25 | 18 | 52.9 | 50.4 |
| **CGT** | 8.3 | 3.2 | 10 | 11 | 8 | 5 | 3 | 13 | 6 | 10 | 8.8 | 8.2 |
| **TTC** | 7.5 | 5.8 | 10 | 18 | 12 | 16 | 9 | 20 | 11 | 17 | 6.1 | 6 |
| **CTT** | 5.3 | 4.2 | 2 | 7 | 16 | 14 | 5 | 3 | 7 | 7 | 3.6 | 5.7 |
| **Others** | 5.8 | 5.8 | 33 | 30 | 26 | 30 | 38 | 28 | 32 | 30 | 8 | 9.7 |

**Supplementary References**

1. Siddiqui A, Kerb R, Weale ME, Brinkmann U, Smith A, et al. (2003) Association of multidrug resistance in epilepsy with a polymorphism in the drug-transporter gene ABCB1. New England Journal of Medicine 348: 1442-1448.

2. Hung C-C, Tai JJ, Lin C-J, Lee M-J, Liou H-H (2005) Complex haplotypic effects of the ABCB1 gene on epilepsy treatment response.6: 411-417

3. Kim DW, Kim M, Lee SK, Kang R, Lee S-Y (2006) Lack of association between C3435T nucleotide MDR1 genetic polymorphism and multidrug-resistant epilepsy. Seizure 15: 344-347.

4. Ebid A-HIM, Ahmed MM, Mohammed SA (2007) Therapeutic drug monitoring and clinical outcomes in epileptic Egyptian patients: a gene polymorphism perspective study. Therapeutic drug monitoring 29: 305-312.

5. Hung C-C, Jen Tai J, Kao P-J, Lin M-S, Liou H-H (2007) Association of polymorphisms in NR1I2 and ABCB1 genes with epilepsy treatment responses.

6. Kwan P, Baum L, Wong V, Ng PW, Lui CH, et al. (2007) Association between< i> ABCB1</i> C3435T polymorphism and drug-resistant epilepsy in Han Chinese. Epilepsy & Behavior 11: 112-117.

7. Ozgon GO, Bebek N, Gul G, Cine N (2007) Association of MDR1 (C3435T) polymorphism and resistance to carbamazepine in epileptic patients from Turkey. European neurology 59: 67-70.

8. Dericioglu N, Babaoglu MO, Yasar U, Bal IB, Bozkurt A, et al. (2008) Multidrug resistance in patients undergoing resective epilepsy surgery is not associated with C3435T polymorphism in the ABCB1 (MDR1) gene. Epilepsy research 80: 42-46.

9. Lakhan R, Misra U, Kalita J, Pradhan S, Gogtay N, et al. (2009) No association of ABCB1 polymorphisms with drug-refractory epilepsy in a north Indian population. Epilepsy & Behavior 14: 78-82.

10. Ufer M, Mosyagin I, Muhle H, Jacobsen T, Haenisch S, et al. (2009) Non-response to antiepileptic pharmacotherapy is associated with the ABCC2-24C> T polymorphism in young and adult patients with epilepsy. Pharmacogenetics and genomics 19: 353-362.

11. Alpman A, Ozkinay F, Tekgul H, Gokben S, Pehlivan S, et al. (2010) Multidrug resistance 1 (MDR1) gene polymorphisms in childhood drug-resistant epilepsy. Journal of child neurology 25: 1485-1490.

12. Grover S, Bala K, Sharma S, Gourie-Devi M, Baghel R, et al. (2010) Absence of a general association between ABCB1 genetic variants and response to antiepileptic drugs in epilepsy patients. Biochimie 92: 1207-1212.

13. Sayyah M, Kamgarpour F, Maleki M, Karimipoor M, Gharagozli K, et al. (2011) Association analysis of intractable epilepsy with C3435T and G2677T/A ABCB1 gene polymorphisms in Iranian patients. Epileptic Disorders 13: 155-165.

14. Hung C-C, Chang W-L, Ho J-L, Tai JJ, Hsieh T-J, et al. (2012) Association of polymorphisms in EPHX1, UGT2B7, ABCB1, ABCC2, SCN1A and SCN2A genes with carbamazepine therapy optimization. Pharmacogenomics 13: 159-169.

15. Ponnala S, Chaudhari JR, Jaleel MA, Bhiladvala D, Kaipa PR, et al. (2012) Role of MDR1 C3435T and GABRG2 C588T Gene Polymorphisms in Seizure Occurrence and MDR1 Effect on Anti-Epileptic Drug (Phenytoin) Absorption. Genetic Testing and Molecular Biomarkers 16: 550-557.

16. Saygi S, Alehan F, Atac FB, Erol I, Verdi H, et al. (2013) Multidrug resistance 1 (MDR1) 3435C/T genotyping in childhood drug-resistant epilepsy. Brain and Development.

17. Shaheen U, Prasad D, Sharma V, Suryaprabha T, Ahuja Y, et al. (2013) Significance of MDR1 gene polymorphism C3435T in predicting drug response in epilepsy. Epilepsy research.

18. Zimprich, F., Sunder-Plassmann, R., Stogmann, E., Gleiss, A., Dal-Bianco, et al.(2004). Association of an ABCB1 gene haplotype with pharmacoresistance in temporal lobe epilepsy. *Neurology,* 63**:** 1087-1089.

19. Seo T, Ishitsu T, Ueda N, Nakada N, Yurube K, et al. (2006) ABCB1 polymorphisms influence the response to antiepileptic drugs in Japanese epilepsy patients. Pharmacogenomics 7: 551-561.

20. Vahab SA, Sen S, Ravindran N, Mony S, Mathew A, et al. (2009) Analysis of genotype and haplotype effects of ABCB1 (MDR1) polymorphisms in the risk of medically refractory epilepsy in an Indian population. Drug metabolism and pharmacokinetics 24: 255-260.

21. Haerian BS, Lim KS, Tan CT, Raymond AA, Mohamed Z (2011) Association of ABCB1 gene polymorphisms and their haplotypes with response to antiepileptic drugs: a systematic review and meta-analysis. Pharmacogenomics 12: 713-725.
